# Oxaliplatin-Induced Tonic-Clonic Seizures

**DOI:** 10.1155/2015/879217

**Published:** 2015-09-30

**Authors:** Ahmad K. Rahal, Phu V. Truong, K. James Kallail

**Affiliations:** ^1^Department of Internal Medicine, University of Kansas-Wichita, 1010 N. Kansas Street, Wichita, KS 67214, USA; ^2^Cancer Center of Kansas, 3243 East Murdock, Suite 300, Wichita, KS 67208, USA

## Abstract

Oxaliplatin is a common chemotherapy drug used for colon and gastric cancers. Common side effects are peripheral neuropathy, hematological toxicity, and allergic reactions. A rare side effect is seizures which are usually associated with posterior reversible leukoencephalopathy syndrome (PRES). A 50-year-old male patient presented with severe abdominal pain. CT scan of the abdomen showed acute appendicitis. Appendectomy was done and pathology showed mixed adenoneuroendocrine carcinoma. Adjuvant chemotherapy was started with Folinic acid, Fluorouracil, and Oxaliplatin (FOLFOX). During the third cycle of FOLFOX, the patient developed tonic-clonic seizures. Laboratory workup was within normal limits. EEG and MRI of the brain showed no acute abnormality. The patient was rechallenged with FOLFOX but he had tonic-clonic seizures for the second time. His chemotherapy regimen was switched to Folinic acid, Fluorouracil, and Irinotecan (FOLFIRI). After 5 cycles of FOLFIRI, the patient did not develop any seizures, making Oxaliplatin the most likely culprit for his seizures. Oxaliplatin-induced seizures rarely occur in the absence of PRES. One case report has been described in the literature. We present a rare case of tonic-clonic seizures in a patient receiving Oxaliplatin in the absence of PRES.

## 1. Introduction

Neoplasms of the appendix are rare [1]. They are found in about 1 percent of appendectomy specimens. Appendiceal cancer is a severe disease, and prognosis depends on the type and size of the tumor and on the metastatic status of diagnosis. Surgery can be curative especially when the tumor is limited to the appendix with no metastasis. Chemotherapy used as adjuvant regimen has yielded encouraging results. Oxaliplatin is an alkylating agent used in colorectal, pancreatic, and gastric cancers. The most common side reactions associated with Oxaliplatin are anorexia, nausea, vomiting, acute dysesthesias triggered or aggravated by cold, and persistent peripheral neuropathy [2]. Oxaliplatin also can cause posterior reversible leukoencephalopathy syndrome (PRES), a rare condition that affects the brain. The diagnosis of PRES should be confirmed by brain imaging. Seizures due to Oxaliplatin are rarely seen without PRES. We present a rare case of tonic-clonic seizures in a patient receiving Oxaliplatin in the absence of PRES.

## 2. Case Presentation

A 50-year-old previously healthy male presented to the emergency room with severe abdominal pain. He denied use of any medications or allergies. He had a family history of breast cancer in his mother and ovarian cancer in his maternal grandmother. He was an ex-smoker and denied use of alcohol or illicit drugs. His physical exam was within normal limits except for diffuse abdominal tenderness. A computed tomography (CT) scan of the abdomen and pelvis showed acute appendicitis. Appendectomy was done and pathology showed mixed adenoneuroendocrine carcinoma (MANEC), most consistent with adenocarcinoma ex-goblet-cell carcinoid, signet ring cell tumor-type, with a maximum dimension of approximately 4.3 cm. He had features of both adenocarcinoma and carcinoid tumor. The patient subsequently underwent a colonoscopy that showed a 7 mm polyp in the descending colon. Biopsy showed a sessile tubular adenoma with no high grade dysplasia. Definitive surgical therapy, a right hemicolectomy, was performed. Lymph nodes showed metastatic goblet cell carcinoid tumor. In the presence of positive nodal disease, it was decided to proceed with adjuvant chemotherapy.

Adjuvant chemotherapy with Folinic acid, Fluorouracil, and Oxaliplatin (FOLFOX) was started. During the third cycle of FOLFOX, the patient developed a witnessed tonic-clonic seizure that lasted approximately 2-3 minutes. He was transferred to the hospital for further evaluation. He denied any previous personal or family history of seizures or strokes. Electrocardiogram (ECG), complete blood count (CBC), complete metabolic panel (CMP), thyroid stimulating hormone (TSH), and urine drug screen were within normal limits. Initial 24-hour urine 5-HIAA was 3.8 mg/24 hr (reference range 0.0–14.9 mg/24 hr) and Chromogranin-A was 3 nmol/L (reference range 0.0–5.0 mg/L). Magnetic Resonance Imaging (MRI) of the brain did not show any acute abnormality. An electroencephalogram (EEG) was normal. The patient was rechallenged with the FOLFOX chemotherapy, however, at the end of the fourth infusion, the patient became unresponsive, his eyes rolled back, and he had tonic-clonic seizures for the second time. Workup including CBC, CMP, and MRI of brain again was negative. His chemotherapy regimen was switched to Folinic acid, Fluorouracil, and Irinotecan (FOLFIRI), thus removing Oxaliplatin from his chemotherapy regimen. After 5 cycles of FOLFIRI chemotherapy, the patient did not develop any more seizures, making Oxaliplatin the most likely reason for his seizures.

## 3. Discussion

Oxaliplatin is a platinum-based antineoplastic agent used to treat colon or rectal cancer that has metastasized [3]. It is often given in combination with other anticancer drugs (Fluorouracil and leucovorin). The primary neurological side effects of Oxaliplatin usually are peripheral neuropathy [4]. Two different types of peripheral sensory neuropathy may occur. An acute neuropathy may occur within hours to 1 to 2 days, which is reversible, with primarily peripheral symptoms that are often exacerbated by cold [5]. Secondly, a more persistent presentation may occur for more than 14 days that often interferes with daily activities. These symptoms may improve in some patients upon discontinuing treatment.

Oxaliplatin can cause PRES, a rare condition that affects the brain [6–8]. Hinchey et al. described a clinicoradiological syndrome characterized by bilateral, reversible, symmetric, vasogenic edema, attributable to a variety of different etiologies, and coined the term “PRES” to describe this syndrome [9]. PRES typically is subacute at onset and characterized by headache and altered mental status. Patients also can present with additional symptoms such as vomiting, cortical blindness, and seizures of occipital origin [9, 10]. Multiple seizures are more common than a single event [9]. PRES is diagnosed with brain MRI; neuroimaging findings typically are characterized by bilateral, reversible, subcortical, symmetric edema, but they also may involve watershed zones and cortical regions posteriorly [10]. Other central and peripheral nervous system disorders associated with Oxaliplatin include loss of deep tendon reflexes, dysarthria, Lhermitte's sign, cranial nerve palsies, fasciculation, and convulsions [11, 12]. The overall incidence of neurotoxicity is 2%-3% [13]. Approaches used to prevent or minimize Oxaliplatin neurotoxicity include stopping and reintroducing Oxaliplatin, dose reduction, and lengthening the duration of infusion. The dose of Oxaliplatin should be reduced for renal dysfunction [14]. Oxaliplatin-induced seizures are reported in less than 1% of the cases, and it usually is associated with reversible encephalopathy syndrome; however, in our case the MRI of brain was normal.

Differential diagnosis in our case included primary seizure disorder, brain pathology, metabolic imbalance, infections, and medication-induced seizures. Brain imaging, including MRI and CT of brain, ruled out pathologic brain disease. EEG ruled out an epilepsy disorder. Laboratory workup, including CMP, CBC, and TSH, was within normal limits ruling out metabolic etiology of the seizures. Also, initial 24-hour urine 5-HIAA and Chromogranin-A were within normal limit ruling out carcinoid syndrome as a cause of the seizures.

Drug-induced seizures are a diagnosis of exclusion. Our patient underwent an extensive workup that ruled out any organic or metabolic cause of his seizures. The only known report of Oxaliplatin-associated seizures was in a patient with gastric cancer receiving Epirubicin, Oxaliplatin, and Capecitabine (EOX). In this case, either Epirubicin or Oxaliplatin was reported as the cause of seizures [15].

The risk of complications from seizures induced by Oxaliplatin may be reduced by increasing awareness of the potential for seizures associated with Oxaliplatin even with normal brain imaging. This risk is probably underestimated. In any case of seizures in a patient taking chemotherapy containing Oxaliplatin, discontinuation of the drug is necessary. However, whether to rechallenge the patient with Oxaliplatin depends on the physician's clinical judgment.

## 4. Conclusion

Oxaliplatin-induced tonic-clonic seizures in a patient without a personal history of pathologic disease or in the absence of PRES are rare. Physicians should be aware that Oxaliplatin can cause seizures even in the absence of PRES.
